# Detection of microbial contamination in chicken meat from local markets in Surabaya, East Java, Indonesia

**DOI:** 10.14202/vetworld.2021.3138-3143

**Published:** 2021-12-20

**Authors:** Dhandy Koesoemo Wardhana, Ajeng Erika Prihastuti Haskito, Muhammad Thohawi Elziyad Purnama, Devi Ayu Safitri, Suwaibatul Annisa

**Affiliations:** 1 Department of Veterinary Sciences, Division of Veterinary Public Health, Faculty of Veterinary Medicine, Universitas Airlangga, Surabaya 60115, Indonesia; 2 Laboratorium of Veterinary Public Health, Faculty of Veterinary Medicine, Universitas Brawijaya, Malang 65151, Indonesia; 3 Department of Veterinary Sciences, Division of Veterinary Anatomy, Faculty of Veterinary Medicine, Universitas Airlangga, Surabaya 60115, Indonesia; 4 Faculty of Veterinary Medicine, Universitas Airlangga, Surabaya 60115, Indonesia

**Keywords:** chicken meat, local markets, microbial contamination, public health, Surabaya

## Abstract

**Background and Aim:**

Chicken meat can be contaminated by microorganisms anywhere in the supply chain, from farm to market, and these microorganisms can be transmitted to humans through direct contact, contact with the environment, and food consumption. The microbial contamination has a serious impact on public health. This study aimed to analyze the microbial contamination of chicken meat sampled from local markets in Surabaya, East Java, Indonesia.

**Materials and Methods:**

A total of 60 samples of fresh chicken meat obtained from 10 traditional markets (six samples per market) were examined for the presence of bacteria. *Staphylococcus aureus*, *Salmonella* spp., and *Escherichia coli* were identified using Gram staining, culturing, and biochemical tests. The most probable number (MPN) method was used to identify *E. coli*.

**Results:**

Most chicken meat samples were positive for *S. aureus* (58.3%), *Salmonella* spp. (48.3%), and *E. coli* (40%). The samples were considered positive for *E. coli* if the MPN value was higher than 1×10^1^ CFU/g.

**Conclusion:**

High microbial contamination was found in all the chicken meat sampled from local markets in Surabaya. Such contamination can lead to foodborne diseases so, proper hygiene and sanitation standards should be followed from slaughterhouses to the end-users.

## Introduction

Poultry meat products are widely consumed worldwide due to their high nutrition level, relatively low-cost production, and short cooking process [1]. Chickens are raised for meat and serve as a low-acid food that is rich in nutrients, phosphorus, other minerals, and B-complex vitamins [2]. Chicken is an excellent source of animal protein with low lipid content and high biological value, which contains all the essential amino acids and unsaturated fatty acids required for a human diet [3]. Chicken meat is widely popular in Indonesia, where an average of 1.11 million tons of it is consumed per year, with production reaching 1.48 million tons per year. Chicken meat is an affordable source of animal protein, yet it is also easily damaged, as the rapid growth of microorganisms can alter its quality [4].

Fresh chicken meat is highly perishable due to its physical and chemical properties.

Its abundance of nitrogenous compounds, lipids, carbohydrates, and vitamins, and its high water holding capacity allow the establishment of a suitable environment for microbial growth [5]. Several pathogenic microorganisms, such as *Salmonella* spp., *Campylobacter* spp., *Staphylococcus aureus*, *Escherichia coli*, and *Listeria* spp., have been found to contaminate poultry [6]. Diseases originating from animals can spread to humans through indirect environmental contact, direct contact, and/or food consumption [7]. Foodborne illnesses worldwide are caused by foodborne microbial pathogens, which endanger food safety due to the risk of ingesting food, primarily animal products, that are contaminated with vegetative pathogens or their toxins. Most of these microbes have zoonotic importance, as they can significantly impact both public health and economic sectors [8]. Rortana *et al*. [9] reported the prevalence of *Salmonella* spp. and *S. aureus* in chicken meat in Cambodia at 40.4% and 46.2%, respectively. A study by Mashak [10] revealed that 16.25% of chicken meat from Alborz, Iran, was positive for *E. coli*. The previous studies also stated that several chicken meat samples from local markets in Indonesia presented microbial contamination, which included *S. aureus* (6.7%) [11], *Salmonella* spp. (85%) [12], and *E. coli* (90.03%) [13]. The contamination of poultry products including raw broiler meat by pathogenic microorganisms, especially bacteria, has become one of the most challenging problems in the food industry worldwide [14]. Poultry meat can be contaminated by different types of microorganisms during operations at meat-processing plants [15]. Contamination can occur during processing, contact with the facility’s equipment (e.g., grinders, belts, and saws), contact with food handlers (e.g., hand contact and knives), and exposure to other environmental sources (e.g., air and water) [16]. The number of bacteria contaminating poultry carcasses may decrease or increase between different processing steps at the plants [17].

Various diseases caused by *S. aureus*, *E. coli*, and *Salmonella* spp. can develop due to a lack of public knowledge and awareness, especially among traders for safety, health, quality, and halal classification, during the process of meat handling and distribution [18].

This study aimed to analyze microbial contamination in chicken meat samples from several local markets in the Surabaya region.

## Materials and Methods

### Ethical approval

Ethical approval for animal research was not required as live animals were not used in this study.

### Study period and location

The study was conducted from July 2020 to September 2020. Chicken meat samples were collected from 10 different local markets of Surabaya. The samples were processed at Veterinary Public Health Laboratory, Faculty of Veterinary Medicine Universitas Airlangga.

### Sampling

In this study, chicken meat was sampled using a purposive sampling method based on several criteria, which included meat selection from sellers with poor sanitation levels, low hygiene for the tools used to cut the meat, and frequent direct contact between the meat and market visitors. Fresh samples (n=60) were collected from 10 different local markets of Surabaya, and they were obtained from the surface of chicken meat. The local markets visited were located in the southern, northern, western, eastern, and central parts of the Surabaya region, and six samples were obtained from each market.

### Isolation and identification of *S. aureus*

*S. aureus* was detected by taking 0.1 mL of samples from the first dilution (10^-1^) for total plate count testing. The sample volume was then inoculated into Mannitol Salt Agar (MSA) (Merck 1.05404.0500) and was incubated at 35°C for 24 h. Yellow colonies growing on the MSA were described as positive *S. aureus*, while red colonies represented other species of *Staphylococcus* [19]. The suspected colonies were identified by mannitol fermentation, Gram staining, a catalase test, and a coagulase test as per standard protocol [20]. Gram staining was conducted aseptically on the identified colonies. Bacterial identification was confirmed using a Nikon Eclipse E100 LED microscope with 1000×. *S. aureus* appeared as Gram-positive coccus bacteria and was purple in color. The presence of this microbe was also validated by positive catalase and coagulase test results [11].

### Isolation and identification of *Salmonella* spp.

Detection of *Salmonella* spp. was carried out by inserting 25 g of each meat sample into 225 mL of Lactose Broth (Merck 1.07661.0500 Darmstadt, Germany) and incubating it at 35°C for 24 h. Then, a 1 mL volume of suspension samples was inoculated into 9 mL of Tetrathionate Broth (TB) (Merck 1.05285.0500 Darmstadt) and was incubated at 35°C for 24 h. One TB loop was taken using an inoculating loop; it was streaked onto Bismuth Sulfite Agar (Merck 1.05418.0500 Darmstadt) and was then incubated at 35°C for 24 h. The typical *Salmonella* colonies were analyzed through Gram staining, lysine iron agar (LIA) media (Merck 1.11640.0500 Darmstadt), triple sugar iron agar (TSIA) media (Merck 1.03915.0500 Darmstadt), Simmons citrate agar (Merck 1.02501.0500 Darmstadt), and a urease test for biochemical identification [21-23]. *Salmonella* spp. isolates showed positive results, as observed in the Simmons citrate agar. The LIA showed a purple slant/purple butt (alkaline), although the butt reaction may have been masked by H_2_S production. The TSIA showed a red slant/yellow butt (alkaline). The presence of *Salmonella* spp. was indicated by negative urease test results [23].

### Identification of *E. coli* through the most probable number (MPN) method

Each sample (25 g) was homogenized in buffered peptone water (Merck 1.07288.5000 Darmstadt, Germany) for the isolation of *E. coli*. One milliliter of the suspension was transferred into 9 mL of brilliant green bile broth (Merck 1.05454.0500 Darmstadt, Germany) with a Durham tube and was then incubated at 45.5°C for 24-48 h. The isolates were suspected to be *E. coli* once the tube was filled with gas. One loop of suspected *E. coli* was taken and streaked onto eosin methylene blue agar (EMBA) (Merck 1.01347.0500 Darmstadt, Germany) and was then incubated at 35°C for 24 h. The suspected colonies from each EMBA were transferred into tryptone water (Merck 1.10859.0500 Darmstadt, Germany) for an indole test. The calculation of MPN for *E. coli* was based on tubes with a positive *E. coli* broth dilution using McCrady’s tables [18].

### Statistical analysis

The data are presented descriptively in percentages and displayed in tables.

## Results

### Prevalence of *S. aureus* and *Salmonella* spp. in chicken meat

Out of the 60 chicken meat samples collected in the present study, 35 *S. aureus* isolates (58.3%) and 29 *Salmonella* spp. isolates (48.3%) were identified using staining, cultural, and biochemical tests. The prevalence of microbial contamination (*S. aureus* and *Salmonella* spp.) is summarized in Table-1.

**Table 1:** Results of bacterial contamination in chicken meat.

Laboratory result	Bacterial contamination	

	*Staphylococcus aureus*	*Salmonella* spp.
Positive sample (n=60)	35	29
% positive sample	58.3	48.3

### Prevalence of *E. coli* in chicken meat

Identification through the MPN method revealed that out of the 60 chicken meat samples, 24 were positive for *E. coli* (40%). The results are summarized in Table-2. The samples were considered positive if the MPN was higher than 1×10^1^ CFU/g, based on the Indonesian National Standard [24].

**Table 2:** Results of *Escherichia coli* in chicken meat.

S. No.	Market code	Mean of MPN value for each market (CFU/G)	Positive sample (n=60)	Percentage
1.	M	>1100	6	100
2.	PG	120	1	16.7
3.	K	<3	0	0
4.	G	3.7	0	0
5.	WK	454	3	50
6.	BB	228	2	33.3
7.	GS	18	0	0
8.	PK	17.4	0	0
9.	B	>1100	6	100
10.	LS	>1100	6	100
Total	24	40

Standard limit MPN=1×10^1^ CFU/G [24]. MPN=Most probable number

## Discussion

Many zoonotic foodborne illnesses are transmitted through chicken meat worldwide, and not only they have a severe impact on public health, but they also impose huge costs on the economy [25]. Some important pathogens associated with these illnesses are *S. aureus*, *Salmonella* spp., *Campylobacter* spp., *Listeria monocytogenes*, and *E. coli* [8]. This study, which investigated chicken meat contamination in relation to foodborne diseases, showed positive results for *S. aureus* (58.3%), *Salmonella* spp. (48.3%), and *E. coli* (40%) in samples derived from local markets in Surabaya.

In particular, the prevalence of *S. aureus* (58.3%) in chicken meat from the 10 Indonesian markets was higher than that found in raw chicken meat sold in Ghana (9.2%), as reported by Pesewu *et al*. [14]. In addition, Kim *et al*. [26] reported that out of 200 chicken meat samples collected in South Korea, 94 were found to be positive (47%) for *S. aureus*. This number was lower compared to the value reported in Savariraj *et al*. [20], where around 66.67% of samples obtained from in retail outlets in Chennai, India, resulted positive for *S. aureus*.

It is hypothesized that *S. aureus* causes infections primarily in humans. This pathogen has been identified as the cause of staphylococcal food poisoning, which is a type of gastroenteritis due to the consumption of meat containing one or more preformed staphylococcal enterotoxins [27].

*S. aureus* transmission may occasionally occur in the community through the consumption or handling of contaminated foods. Severe foodborne diseases caused by this bacterium have been explicitly demonstrated to develop in healthy individuals for years [28,29]. The contamination of meat with *S. aureus* occurs as a result of poor hygiene during slaughter and handling, and also because of the poor quality of the water used during meat processing. It can occur during several phases throughout the process, including meat production, slaughter, handling, and marketing to consumers [30].

The zoonotic transmission of *S. aureus* from poultry has typically been investigated in regard to antimicrobial resistance. This is considered a crucial issue in the poultry industry due to its impact on public health and because it poses a challenge to medical and veterinary professionals globally [31].

Out of the 60 chicken meat samples examined in this study, 29 (48.3%) resulted positive for *Salmonella* strains. This prevalence of contamination was higher than that found in studies conducted in other countries, which have reported a prevalence of 10.8% [14] for *Salmonella* in chicken meat sold in Korle Gonno, Accra (Ghana), and of 9.5% [32] in that sold in Chongqing, China.

*Salmonella* is a significant zoonotic pathogen that causes human gastroenteritis outbreaks, with occasional cases worldwide [33]. *Salmonella* infections are frequently correlated with the ingestion of contaminated animal products [34]. Chicken products and raw, or undercooked, eggs have been identified as potential transmitters of this bacterium in humans based on previous studies and epidemiological investigations [35,36]. *Salmonella enteritidi*s*, Salmonella typhimuriu*m*, Salmonella enteritica* serotype Newport, *Salmonella enterica* serova*r* Infantis*, Salmonella* serovar Montevideo, *Salmonella enterica* subsp. *enterica* Serovar Heidelberg*, Salmonella enteritica* serotype Senftenberg, and *Salmonella enteritica* serova*r* Schwarzengrund are serotypes regularly isolated in different countries, frequently from chicken carcasses, other cut parts, and by-products [37]. The most prevalent serotypes have been shown to cause enteritis and are responsible for foodborne infections [38].

Out of the 60 chicken meat samples investigated in this study, 24 (40%) resulted positive for *E. coli* contamination, with a number of germs exceeding 1×10^1^ CFU/g (Table-2). Ayodele *et al*. [39] claimed that no *E. coli* was detected in chicken meat from a retail market in Ibadan, Nigeria, while Rahman *et al*. [40] reported that 63.5% of the chicken meat samples were collected in Bangladesh tested positive for *E. coli*. Rahmahani *et al*. [41] conducted a study in traditional markets in Surabaya, East Java (Indonesia), and discovered that 96.7% of domestic chicken also contained this bacterium. This high detection rate indicates that chicken meat received from market was unsafe for human consumption, based on the prescribed limits [42].

Animal-derived food is frequently contaminated with *E. coli* bacteria, which are found in the typical intestinal flora of animals and humans. This bacterium may contribute to prevent the development of harmful organisms in the gastrointestinal tract. Certain strains of *E. coli* frequently become pathogenic due to the pathogenic and virulent properties of several genes included in transmissible genetic elements [43]. Diarrhea, urinary tract infections, sepsis, and meningitis can be caused by pathogenic *E. coli* strains [44].

These infections are likely to be spread by workers through processing equipment, such as blades and cutting boards, and poor hygiene and sanitation [45]. Contamination can occur during the slaughtering process through direct contact with the slaughterhouse workers’ hands, water, and equipment [46]. The high contamination levels observed in this study may be due to the manual slaughter and butchering of animals at slaughterhouses and markets. Indeed, this contaminating flora originates mainly from the animals’ skins, from the carcasses handled, and from direct contact with dirty work areas during slaughtering operations [47]. The presence of bacteria in meat-processing equipment and associated surfaces may also contribute to meat contamination at the markets [48].

The poultry industry is a vertically integrated system of production, processing, and distribution. In the broiler farming business, this system enables producers to integrate various biosecurity and sanitation techniques, housing technology, and feeding regimens to improve food safety [26]. These emphasize the importance of providing adequate training in food hygiene to those who handle this type of food, to avoid unsafe practices (such as cross-contamination or insufficient cooking), and thus reduce the risks for consumers [29]. Cross-contamination can be avoided by maintaining high hygiene and sanitation standards during food processing [49].

## Conclusion

From the data acquired in this study, it can be concluded that high microbial contamination was present in all the 60 chicken meat samples collected from local markets in Surabaya. Specifically, the samples were contaminated by *S. aureus* (58.3%), *Salmonella* spp. (48.3%), and *E. coli* (40%). Therefore, it is recommended to implement the hazard analysis critical control points system and food hygiene practices during food handling and processing. Further research is required to fully comprehend the dynamics of this type of contamination occurring within the food chain.

## Authors’ Contributions

DKW, AEPH, MTEP, DAS, and SA: Carried out the main research works. MHE: Performed the analysis of data. DKW, DAS, and SA: Prepared the manuscript. DKW: Revised the manuscript. All authors have read and approved the final manuscript.
